# COVID‐19 induced bradyarrhythmia and relative bradycardia: An overview

**DOI:** 10.1002/joa3.12578

**Published:** 2021-06-14

**Authors:** Steven Douedi, Anton Mararenko, Abbas Alshami, Mohammed Al‐Azzawi, Firas Ajam, Swapnil Patel, Hani Douedi, Dawn Calderon

**Affiliations:** ^1^ Department of Internal Medicine Jersey Shore University Medical Center Neptune NJ USA; ^2^ Department of Cardiology Jersey Shore University Medical Center Neptune NJ USA; ^3^ Department of Cardiology Community Medical Center Toms River NJ USA

**Keywords:** arrythmia, bradyarrhythmia, bradycardia, coronavirus, COVID‐19

## Abstract

Novel coronavirus 2019 (COVID‐19) has been the focus of the medical community since its emergence in December 2019 and has already infected more than 100 million patients globally. Primarily described to cause a respiratory illness, COVID‐19 has been found to affect almost every organ system. Bradycardia is a newly recognized ramification of COVID‐19 that still has unknown prognostic value. Studies have shown an increase in the incidence of arrhythmias, cardiomyopathies, myocarditis, acute coronary syndromes, and coagulopathies in infected patients as well as an increased risk of mortality in patients with preexisting cardiovascular disease. While the pathogenesis of bradycardia in COVID‐19 may be multifactorial, clinicians should be aware of the mechanism by which COVID‐19 affects the cardiovascular system and the medication side effects which are used in the treatment algorithm of this deadly virus. There has yet to be a comprehensive review analyzing bradyarrhythmia and relative bradycardia in COVID‐19 infected patients. We aim to provide a literature review including the epidemiology, pathogenesis, and management of COVID‐19 induced bradyarrhythmia.

## Abstract 2

Bradycardia is a newly recognized ramification of the COVID‐19 that still has unknown prognostic value. Studies have shown an increase in the incidence of arrhythmias, cardiomyopathies, myocarditis, acute coronary syndromes, and coagulopathies in infected patients as well as an increased risk of mortality in patients with preexisting cardiovascular disease. While the pathogenesis of bradycardia in COVID‐19 may be multifactorial, clinicians should be aware of the mechanism by which COVID‐19 affects the cardiovascular system and the medication side effects which are used in the treatment algorithm of this deadly virus.

## 1 INTRODUCTION

The emergence of the novel coronavirus 2019 (COVID‐19) resulting in pulmonary disease, or better known as severe acute respiratory syndrome coronavirus 2 (SARS‐CoV‐2), has presented the world with an unprecedented medical challenge.^1^, ^2^ The virus’ high infectivity, long incubation period, and ability to be transmitted even while the host is asymptomatic contribute to its rapid spread. Currently, to date, there have been over 108 million reported cases and well over 2 million deaths worldwide.^3^ From the moment it was officially identified in Wuhan in December 2019, the rapid spread of the virus has outpaced our ability to develop a thorough understanding of the pathophysiology as well as clinical ramifications of this disease.^2^ As the total cases of COVID‐19 have increased and advancements in research have progressed, more of the disease becomes known including the various manifestations and complications.

While primarily defined as a pulmonary disease, various multiorgan complications have been identified including the cardiovascular system. Cardiovascular involvement has been seen with conditions, such as myocarditis, cardiogenic shock, acute coronary syndrome, and various arrhythmias.^4^, ^5^ There has also been an increased incidence of bradycardia and relative bradycardia in patients with COVID‐19 infection.^5^, ^6^, ^7^ These cardiac complications have also been observed in other viral and bacterial infections such as influenza A, typhoid fever, Legionnaire's disease, and *Chlamydia psittaci* infections; however, may play a larger role and have increased incidence in COVID‐19.^8^, ^9^ There are several plausible mechanisms for bradycardia that may play a role in patients with COVID‐19 and other infections. The exact mechanism can also vary between the clinical setting and administered treatments or medications. We aim to provide an overview of the literature that discusses the relationship between bradycardia and patients with COVID‐19 exploring the epidemiology, outcomes, and challenges associated with management.

## 2 CARDIOVASCULAR COMPLICATIONS AND EPIDEMIOLOGY IN COVID‐19

COVID‐19 is known to cause a wide array of extrapulmonary manifestations, especially on the cardiovascular system. Studies have shown an increase in the incidence of arrhythmias, cardiomyopathies, myocarditis, acute coronary syndromes, and coagulopathies in infected patients as well as an increased risk of mortality in patients with preexisting cardiovascular disease.^5^, ^10^, ^11^ It has been suspected arrhythmias in COVID‐19 may arise due to electrolyte abnormalities, acidosis, and hypoxemia although the exact cause is still unknown.^5^, ^10^ Wang et al reported the incidence of arrhythmias in COVID‐19 infected patients to be as high as 16.7%.^12^

In regard to bradyarrhythmia, some studies have reported a significantly higher incidence in hospitalized patients. Hu et al reported about 33% of patients with severe COVID‐19 infection developed sinus bradycardia, all of which improved after treating the viral infection.^13^ Capoferri et al reported 110 hospitalized COVID‐19 patients in which 36% developed relative bradycardia and of the patients with a fever (temperature >38.3℃) 56% developed relative bradycardia.^7^ Ikeuchi et al reported similar findings where relative bradycardia was defined as a characteristic finding in COVID‐19.^14^ While most studies have reported improvement in bradycardia with medical management, some cases of severe bradyarrhythmia and complete heart block requiring intervention have also been reported.^15^, ^16^

One major review covering cardiac complications in hospitalized COVID‐19 patients reported that 8% of patients (n = 51) developed a significant sinus bradycardia while another 8% developed complete heart block involving either first degree (n = 18) or second degree (n = 21) atrioventricular block.^17^ Unfortunately, there was no clear description of how many patients underwent intervention as well as their response to therapy. Similarly, Chinitz et al reported seven patients that developed a worsening of or new‐onset bradycardia in the setting of COVID‐19 infection.^16^ Of the seven patients observed, three received a temporary pacemaker while the other four underwent permanent leadless pacemaker placement. These patients were severely ill as only 2 had survived 3 months posthospitalization.

Zhou et al presented a report of 97 previously admitted COVID‐19 survivors of which cardiac abnormalities were found in 42.3% of patients.^18^ Sinus bradycardia was present in 29.9% of patients, atrial fibrillation 1.0%, and bradycardia with a heart rate of less than 50 beats per minute in 7.2% of patients.^18^ Additionally, 6.2% of patients were found to have troponins elevated above baseline, however, echocardiography or cardiac magnetic resonance imaging after discharge revealed no acute findings.^18^ Similarly, Chen et al reviewed the extent of cardiovascular involvement in 54 patients with significant COVID‐19‐related complications.^19^ The most frequently reported arrhythmia was noted to be sinus tachycardia in roughly 38 of the patients followed by an atrioventricular block in 2. Furthermore, 6 of the patients, 5 of which were critical, developed pericardial effusions and 3 had new‐onset heart failure.^19^

This data was further supported by another review reported by Kunal et al that reported 108 patients, largely males, with an average age of 51 years.^20^ The most common comorbidities included hypertension (38%) and diabetes mellitus (32.4%). The most common cardiac arrhythmia included sinus tachycardia in 16.9% (n = 18) of patients followed by first degree AV‐block in 4.6% (n = 5) and ventricular tachycardia or fibrillation in 1.8% (n = 2).^19^ The most severe complications included cardiac injury in 25.9% of patients and cardiogenic shock, heart failure, and acute coronary syndrome in 3.7% of patients in each category.^19^

One of the largest and most comprehensive reports of arrhythmias associated with COVID‐19 is provided by Coromilas et al.^21^ Their report includes 4526 patients encompassing 12 countries across four continents that were confirmed to have COVID‐19 by nasopharyngeal PCR from January to June 2020. Of the 4526 patients, 827 of them were found to have a cardiovascular arrhythmia secondary to COVID‐19.^21^ Of these, 599 were found to have atrial fibrillation, atrial flutter, or supraventricular tachycardia, 164 were found to have ventricular tachycardia or ventricular fibrillation, and 172 developed a form of bradyarrhythmia, which included either bradycardia (12.8%), AV block (8.6%), or a pause greater than 3 seconds (1.2%).^21^ Tachycardias were most common among patients in Europe (64.5%), South America (66%) and North America (62.1%), but significantly less common in patients from Asia (34.1%).^21^ Interestingly, bradyarrhythmias, including bradycardia, AV block, and pauses >3 seconds, were much more common in patients from Asia (43.2%) versus those from Europe (18.8%), North America (21.8%), and South America (22%); however, the exact mechanism by which different races were affected has not fully been studied and requires further investigations.^21^

Although tachycardia has been reported to be more common in the setting of COVID‐19 infection, bradycardia has been suspected to be associated with worse outcomes and more severe disease and inflammation.^6^ The onset of bradycardia was found to typically range between the first and third week of infection and commonly be transient in nature.^6^, ^22^ Relative bradycardia has also been proposed to increase in incidence with age, most reported cases occurred in patients over the age of 65.^23^

## 3 MECHANISM OF CARDIAC INVOLVEMENT

The impact of SARS‐CoV‐2 on the myocardium and connection with bradycardia in patients with COVID‐19 is likely multifactorial that varies with disease severity as well as clinical setting. While the exact mechanism remains unknown, there are several physiological pathways that can provide a partial explanation. Prior to exploring the possible mechanisms, we must consider how the virus invades the host and why we have seen several unique presentations of the virus in a host. One of the most popular theories stems to the association of coronavirus and the angiotensin‐converting enzyme 2 (ACE2) receptors.^24^ It is likely that coronavirus has an inherent ability to invade the myocardium. This theory first gained traction after an analysis that was performed by Xu et al which demonstrated that the novel coronavirus isolated in 2019 and the original SARS‐CoV share a common origin in the virus initially found to be carried by bats, the bat coronavirus HKU9‐1.^25^ The basis of this theory is that these viruses share a similar protein based on 3D rendered images and are known to have strong binding affinities to the human angiotensin‐converting enzyme 2.^25^ Therefore, the logic is that if a host overexpresses ACE2 receptors then they are more susceptible to COVID‐19 infection.

To better understand how the novel coronavirus enters a host, Han et al designed a study where they characterized different organ tissues and their expressiveness of the ACE2 receptor.^26^ Their research had demonstrated that ACE2 protein, based on scRNA sequencing, is vastly enriched and proliferative in the enterocytes of the small intestine, renal tubules, heart cells, arterial cells, and alveolar epithelial cells.^26^ This characterization may help explain the unique presentations of the virus as well as why critically ill patients can develop multiorgan failure.^12^

Like other bacterial and viral infections, COVID‐19 can induce bradycardia by increasing the production and release of inflammatory cytokines. Capoferri et al found in their studies that of the inflammatory markers, interleukin‐6 had the strongest correlation with relative bradycardia and other arrhythmias.^7^ Interleukin‐6 is a significant inflammatory marker associated with the cytokine storm seen in COVID‐19 infection and has been reported to have direct impact on the sinoatrial node causing increased vagal tone and minimized heart rate variability ultimately leading to relative bradycardia.^27^, ^28^, ^29^ In addition to these hypotheses, as the disease course worsens arrhythmias are likely worsened by the hypoxia and multiorgan failure, resulting in electrolyte derangements and buildup of toxic metabolites.^5^, ^10^

While the exact mechanism by which bradycardia occurs in COVID‐19 and other infectious processes is still unknown, there have been a number of studies attempting to further evaluate this phenomenon.

## 4 MEDICATION ASSOCIATION WITH BRADYCARDIA

At the start of the pandemic, there were several anecdotal examples that claimed antimicrobials and immunomodulators had efficacy against COVID‐19. Initially, azithromycin and hydroxychloroquine were commonly administered to patients with COVID‐19.^2^ It was not until later that we learned through experience the limited efficacy of these agents. Adding insult to injury, these are medications are known to induce arrhythmias.^30^ Azithromycin, a macrolide antibiotic, works by pausing protein production by inhibiting the 50S ribosomal subunit is commonly used to treat community‐acquired pneumonia. A notorious side effect is the prolongation of the QTc interval, a pathway vital to the proper functioning of the ventricles.^30^ Hydroxychloroquine, a medication often used to treat malaria and rheumatoid arthritis with an unknown mechanism of action was also used in COVID‐19 infection.^2^ Shortly after widespread use, the medical community quickly learned that hydroxychloroquine is also associated with bradyarrhythmia, ventricular escape arrhythmias, and even complete heart block.^31^, ^32^, ^33^

Remdesivir is another commonly used medication that found to have therapeutic benefit against COVID‐19 by halting viral production and is associated with a decreased hospital stay in patients presenting with hypoxia.^34^ Although now being a part of several COVID‐19 treatment algorithms, remdesivir was also found to be associated with bradycardia and reported to affect the QTc interval.^35^, ^36^

## 5 MANAGEMENT OF BRADYCARDIA IN SETTING OF COVID‐19 INFECTION

Cardiovascular associations, predominantly arrhythmias, and complications, have become better understood as the medical community had the opportunity to evaluate the disease course of COVID‐19.^15^, ^16^ Patients that developed a form of cardiac arrhythmia were treated with the appropriate, standard of care modalities. If a medication used in the treatment of COVID‐19 was suspected to be the cause of significant or symptomatic bradycardia, the medication was discontinued. In cases where the cause was unknown, amiodarone, and sotalol were the most frequently used antiarrhythmic medications and pacemakers played a role in therapy in some patients.^21^ Bradycardia poses a significant risk to survival as cases of heart block are on the rise. Currently, there are no clear guidelines on the management of bradycardia in the setting of COVID‐19 aside from the standard treatment in non‐COVID patients.

## 6 CONCLUSION

The global impact of COVID‐19 is immeasurable, and the virus has strained the medical community worldwide. In a field where the action is guided by knowledge and understanding of the pathophysiological basis of disease, one of the most difficult aspects of treating COVID‐19 is the very limited information we have. As we continue to learn more about the presentation and complications of the disease, we begin to understand that COVID‐19 is best characterized by multiorgan involvement. Bradycardia is a newly recognized ramification of the disease that still has unknown prognostic value. Further collaboration among the medical community is vital so that we may gain a better understanding of outcomes as well as the need for early intervention. Most importantly, larger studies and data on expressing outcomes and progressions of patients that developed bradycardia in the setting of COVID‐19 infection are still needed. With close collaboration among the medical and scientific community, we will gain a better understanding of the disease course as well as indications for therapy.

## CONFLICTS OF INTEREST

Authors declare no conflict of interests for this article.

## Data Availability

The authors declare that data supporting the findings of this study are available within the article.
